# Supplementary material for: Are carbon emissions trading and green financial instruments synergistic? -Comprehensive quantitative research based on content analysis
Source: PLoS One. 2024 Mar 7;19(3):e0298601. doi: 10.1371/journal.pone.0298601 (PMC10919601; doi:10.1371/journal.pone.0298601)
Supplement: S1 Data — (ZIP) [file pone.0298601.s001.zip › Data/地级市Z_F工作报告.zip!/║╙─╧/─╧╤⌠╩╨/└┤╫╘╣1⁄2╓┌║┼í╛┬φ┐╦╩2╛▌═°í┐_2013.docx]

各位代表

现在我代表市人民政府向大会作工作报告请予审议并请各位政协委员和其他列席人员提出意见。

折叠去年工作回顾

(一)、奋力稳增长调结构经济发展逆势而进。有效化解经济下行压力主要经济指标增速高于全省平均水平全市经济在逆境中实现平稳较快发展。全市生产总值2367亿元增长10.2%。全社会固定资产投资1819亿元增长23.2%。社会消费品零售总额1076亿元增长15.7%。规模以上工业增加值855亿元增长16.3%。财政总收入181.9亿元增长17.5%其中地方公共财政预算收入103.7亿元首次迈上百亿元台阶增长19%。公共财政预算支出386.6亿元增长24.8%。二三产业占生产总值比重达到82%。六大战略支撑产业对规模以上工业增长的贡献率达到72%三大战略新兴产业增速高于规模以上工业增速12.1个百分点。金融机构新增贷款194亿元余额达到1113亿元突破千亿元大关。全年共接待游客2960万人次实现旅游综合收入152亿元。文化产业增加值达到46亿元增长10.8%。自主创新能力不断增强。高新技术产业增加值达到75亿元增长25.5%。新创国家级品牌22个、省级品牌110个南阳防爆集团获得省长质量奖实现我市零的突破。

(二)、全力抓好两大"国字号"工程综合带动效应持续显现。举全市之力积四年之功我们践行承诺办成了一届精彩圆满、富有特色、惠及民生、影响深远的农运盛会。通过农运会筹办中心城区"三桥三路"、七大出入市口改造、邕河治理、体育场馆、宾馆酒店等一大批基础设施和民生项目如期建成城市功能加速提升"两争一迎"四级联动绿亮美净畅工程卓有成效城乡面貌焕然一新。全市人民用双手和智慧创造了农运辉煌用担当和拼搏熔铸了农运精神用热情和奉献展示了南阳形象。南水北调中线工程服务保障有力干渠开挖成型70%以上配套工程全面启动农村外项目建设稳步推进渠首区地震安全工程顺利完成移民后续稳定发展工作扎实有效库区及上游地区经济社会发展规划、水污染防治和水土保持规划全面实施库区水质保持在Ⅱ类以上。南阳在服务保障中线工程中表现出的大局意识和奉献精神赢得了社会各界的充分肯定和高度评价。

(三)、统筹"三化"促转型五区建设迈出新步伐。产业集聚区建设提速增效。集中开工项目201个总投资882亿元。全年完成固定资产投资1076亿元对投资增长的贡献率达到72%规模以上工业实现主营业务收入1638.8亿元占全市的63.5%。7个产业集聚区规模以上工业营业收入超百亿元。西峡产业集聚区晋升为省级高新区新能源产业集聚区被评为全省先进产业集聚区。淅川、新野、社旗、邓州等产业集聚区集群发展态势初步显现。中心城区规划建设实现新突破。《南阳市土地利用总体规划(2006-2020)》获国家批准争取新增城镇工矿用地指标97平方公里。建成区面积达到120平方公里。南阳新区扎实起步合同(协议)引资额达105亿元。南阳高新区创建国家创新型特色园区启动实施。官庄、鸭河工区规划建设步伐加快招商引资和项目建设进展迅速。县城和小城镇建设快速推进亮点纷呈。新型农村社区稳步推进。选点布局规划顺利完成100个试点全部开工建设已累计投入资金126亿元建成住宅2.5万余套。粮食主产区和农业产业化集群建设成效明显。建成高标准粮田130万亩粮食总产达122.4亿斤实现九连增跻身全国粮食生产先进市。20个农业产业化集群规划建设稳步推进。西峡香菇、猕猴桃产区获评全国出口食品农产品质量安全示范区。新野县成功创建国家现代农业示范区。农村土地适度规模经营面积达到202万亩。高效生态经济示范市启动建设。《南阳市建设中原经济区高效生态经济示范市总体方案》出台实施。我市被列为第五批全国生态文明建设试点地区成为全省唯一入选的省辖市。一批节能减排和循环经济项目通过国家验收认定。单位生产总值能耗和主要污染物排放量持续下降。成功创建全国绿化模范城、白河国家湿地公园和兰湖省级森林公园。

(四)、举市开放抓项目发展后劲明显增强。开放招商成果丰硕。成功组织参加"百名闽商进南阳"、"浙商南阳行"和全省承接产业转移等招商活动。全年实际利用外资4.2亿美元到位省外资金420亿元分别增长22%、70.3%。实施央企合作项目41个完成投资118亿元。与北京、天津对口协作进一步深化。新缔结韩国春川、罗马尼亚斯洛博齐亚2个国际友好城市。项目建设成效显著。46个省重点项目、249个市重点项目分别完成投资125亿元、462亿元分别占年度计划的138%、135%。宁西铁路复线、蒙西至华中铁路、河南油田特种蜡生产基地、天池抽水蓄能电站等重大项目前期工作进展顺利。南阳机场扩建、"3+1"快速通道等交通项目全面完成内邓高速主体完工三淅高速开工建设新建农村公路400余公里改造危桥6000余延米。

(五)、加大投入惠民生和谐社会建设扎实推进。"十项重点民生工程"顺利完成。财政用于民生支出285亿元占财政支出的73.8%。城镇居民人均可支配收入、农民人均纯收入分别增长13%、14.4%。央视2012年度公众调查公布南阳居民幸福感居全国地级城市榜首。就业形势总体稳定。实现各类就业25万人城镇登记失业率控制在4%以内"零就业家庭"动态归零。大力实施全民技能振兴工程完成各类培训30.6万人。社会保障水平不断提高。城乡居民社会养老保险参保人数达529.6万人居全省第一位城镇基本医疗保险制度不断完善新农合参合率达到99%。社会救助工作持续扩面提标困难群众得到更多实惠。社会事业快速发展。医药卫生体制改革扎实推进中医药事业健康发展。顺利通过省级卫生城市复核验收。学前教育三年行动计划稳步实施义务教育发展均衡性增强高考一本上线率跃居全省第二南阳农业职业技术学院获省政府批复张仲景医学院筹建进展顺利。人口计生工作保持全省先进位次。全国全民健身示范市创建工作扎实推进。12.1万农村贫困人口实现脱贫新解决63.7万人饮水安全问题。开工建设各类保障性住房2.8万套完成农村危房改造4.2万户。文化惠民工程深入实施公共图书馆、文化馆、文化站和13家博物馆、纪念馆实现免费开放建成农家书屋1488个完成3104个广播电视村村通项目。和谐社会建设稳步推进。安全生产形势良好。连续四年被评为省"食品安全优秀城市"。信访工作不断加强。综合打击效能全省第一获得"中原卫士杯"竞赛全省第一名社会治安秩序持续好转特别是农运安保工作实现了涉会"零发案、零事故、零失误、零投诉"目标荣获全省平安建设优秀省辖市称号人民群众安全感明显提升社会大局和谐稳定。

同时精神文明、民主法制和政府自身建设进一步加强。深入开展"转、正、提"活动政府效能和公务员素质明显提升。清理整顿了一批行政审批和行政事业性收费项目6个县级公共资源交易中心正式运行完成了事业单位清理规范工作。坚持依法行政自觉接受人大、政协监督坚持定期向人大报告工作向政协通报情况办理省、市人大建议、政协提案665件办复率100%满意或基本满意率达90%以上。积极推进军民融合式发展成功创建全国双拥模范城。工商、质监、税务、海关、检验检疫、审计、统计、民族宗教、史志、档案、外事侨务、邮政、通信、电力、残疾人、农机、气象、地震、人防、对台、国防教育、驻外办事机构等工作都取得了新成绩。

各位代表!过去一年形势严峻复杂挑战非同寻常每一项成绩的取得无不凝聚着全市人民的心血和汗水见证着方方面面的大力支持。

在此谨向全市各条战线上的工人、农民、干部、知识分子和广大建设者向人大代表、政协委员向各民主党派、工商联、无党派人士和人民团体向离退休老领导、老同志向驻宛部队、武警官兵、公安民警向所有关心支持南阳发展的社会各界人士表示崇高的敬意和衷心的感谢!

各位代表!成绩来之不易经验弥足珍贵。实践中的主要体会有一是必须着眼全局谋发展。跳出南阳看南阳在全省全国大格局中审视谋划推动南阳发展全力抓好两大"国字号"工程在站位全局、服务保障大局中加快自身发展。二是必须抓好载体促发展。抓好产业集聚区、农业产业化集群、商务中心区和特色商业区建设深入开展"两争一迎"精心筹办好全国农运会等节会充分发挥各类载体平台的综合带动作用促进整体工作快速提升。三是必须开放创新激活力。强力推进对外开放和改革创新激活内力广借外力强化创新驱动不断增强发展活力。四是必须勇于担当强动力。弘扬敢于担当的移民精神和农运精神拉高工作标杆严格工作标准强化工作落实履职尽责负重拼搏奋发有为增强工作动力争创一流业绩。五是必须改善民生聚民心。把改善民生贯穿到各项工作的始终坚持在发展中改善民生在改善民生中赢得民心、集中民智、凝聚民力。

我们清醒地认识到经济社会发展中还存在一些突出矛盾和问题一是发展速度还不够快。生产总值增速等经济指标与预期还有一定差距晋位升级的压力较大。二是结构性矛盾突出。三次产业结构不合理规模以上工业在二产中比重低城镇化率、中心城市首位度和经济外向度低于全省、全国平均水平大项目、大企业少产品多处于产业链和价值链的低端。三是改善民生任务艰巨繁重。民生和社会保障欠账多、基础差群众上学难、看病难等民生问题依然突出。四是发展环境有待改善。政务环境、投资环境、市场环境、法治环境不优一些部门官僚主义、形式主义问题仍然突出一些领域不正之风还比较严重一些政府工作人员的全局意识、责任意识、服务意识不强。对此我们一定高度重视认真解决。

折叠今年政府工作总体要求和目标任务

今年是深入贯彻落实党的十八大精神的开局之年也是建设中原经济区高效生态经济示范市的关键之年。当前我市发展面临的形势更为复杂严峻国际金融危机和主权债务危机深层次影响不断显现世界经济低迷态势仍将延续。国内经济增长动力不足扩大有效需求面临不少制约因素。外部不利因素和我市结构性矛盾相互叠加造成我市经济出现明显下滑的严峻局面矛盾的深刻性也决定了这种局面难以在短期内得到根本改变推动经济持续较快发展、实现转型崛起的困难和压力很大。同时我们更要看到积极因素和有利条件我国发展仍处于重要战略机遇期经济发展的基本面没有发生根本改变国际国内产业按照比较优势梯次转移的趋势仍在持续中原经济区规划全面实施高效生态经济示范市建设加快推进两大"国字号"工程的综合带动效应持续显现特别是近年来我市的发展基础越来越扎实、发展战略和思路越来越清晰这都为我市长期向好发展提供了有利环境和诸多机遇。我们既要把困难看得大一些把问题看得重一些始终保持清醒忧患更要坚定信心凝心聚力开拓奋进努力为实现全面建成小康社会扎实开局。

今年政府工作的总体要求是认真贯彻落实党的十八大精神紧扣主题主线以提高经济增长的质量和效益为中心以建设中原经济区高效生态经济示范市统揽全局坚持工业强市战略突出抓好扩大开放、新型城镇化、产业集聚区、环境建设四个重点深入实施"四个带动"推进"三化"协调、"四化"同步发展奋力实现南阳绿色崛起。

按照到2020年与全省、全国同步建成小康社会和城乡居民收入翻番的总体目标结合当前形势和我市实际今年经济社会发展的主要预期目标是生产总值增长10%以上规模以上工业增加值增长16%以上地方公共财政预算收入增长11%全社会固定资产投资增长25%社会消费品零售总额增长16%进出口总额增长15%实际利用外资增长10%实际到位省外资金增长25%城镇居民人均可支配收入增长9%农民人均纯收入增长9%以上人口自然增长率控制在6.5%。以内居民消费价格指数控制在3.5%以内节能减排完成省下达目标。

实现上述要求和目标做好今年工作必须把握好以下几点一是着力强基础。加强基础设施和基础产业建设针对薄弱环节抓投入、上项目、补短板增强实现科学发展的基础支撑能力。二是着力创优势。集中培育产业优势巩固扩大生态优势加快构建体制优势着力营造环境优势以创造优势增强综合竞争力。三是着力破瓶颈。把改革创新作为解决深层次矛盾、加快转变经济发展方式的治本之策强力推进重点领域和关键环节改革构建有利于科学发展的体制机制坚持超常规、不违规创新方式方法努力破解土地、资金、人力资源等要素制约。四是着力惠民生。牢牢把握改善民生这一根本目的把推动经济发展与保障和改善民生有机结合起来千方百计办好涉及民生的实事好事让发展成果更多、更公平地惠及于民。

折叠今年重点抓好以下七个方面工作

(一)、加快推进开放招商提升开放型经济水平。

开放招商"一举求多效"。着力构建举市开放体制扎实开展"开放型经济发展推进年"活动坚持招商引资和项目落实"两手抓"政府引导与企业主导相结合积极承接产业转移形成全方位、多层次、宽领域的开放格局。一是突出产业招商。进一步明晰中心城区和各县市的主导产业围绕壮大支柱产业、培育龙头企业和推进产业集聚创新招商方式分产业制定产业链图谱、招商路线图和专项行动方案锁定目标企业瞄准产业链上的关键环节提高推介水平有针对性地开展纵向延链和横向集聚招商变招商选资为选商引资力争在重点产业引进一批高层次的战略支撑项目、龙头企业和协作配套企业。在持续推进工业领域开放的同时重视服务业、农业和科技、医疗、文化、教育等社会事业领域的开放招商。二是突出抓好重点战略合作伙伴、重点区域招商。全力抓好以浙商、闽商项目为重点的招商引资签约项目的落地投产。突出招大引强瞄准国内外500强和行业先锋企业建立高水准的合作名单目录加强与重要合作伙伴的对接联系深化与中石化、富士康的战略合作积极推动与华能集团、首钢控股、雨润集团等大企业的合作。加强与北京的对口协作重点推动与中关村的合作力争在承接高新科技产业转移和孵化上取得突破。继续抓好长三角、珠三角、环渤海等经济发达地区的招商。三是打造、用好开放载体。培育产业集聚区、商务中心区、特色商业区等承接载体建立中心城区招商引资统一协调机制做好南阳卧龙综合保税区申报工作力争新增两家省级经济开发区。巩固扩大第十届玉雕节暨玉文化博览会、月季文化节成果精心组织筹办第十一届张仲景医药科技文化节和中西部协作区联席会议等活动积极参加国家、省组织的重大招商活动力争取得丰硕成果。打造招商引资全程服务平台加快招商引资信息处理和受理中心、联审联批中心、投诉权益保护中心等服务平台建设。健全招商引资绩效考评、重大项目进展情况督查通报等机制努力提高签约项目履约率、开工率、建成投产率。四是着力扩大外经外贸。实施"科技兴贸"和品牌发展战略鼓励企业走出去强化出口基地建设优化出口商品结构抓好石油钻采、汽车零部件、食用菌、花卉、工艺品等产品出口。着力引进出口型项目大力发展加工贸易实施外贸孵化成长工程力争出口超5000万美元的企业达到4家超千万美元企业达到30家以上。五是优化发展环境。优化环境"一优带百通"。把解决发展环境问题作为促进开放、保障发展的当务之急痛下决心优化发展环境打造最具吸引力的投资热土和创业乐园。高位启动全市环境优化建设活动在优化政务环境、投资环境、市场环境、法治环境等方面研究出台过硬措施严厉查处一批典型案例。继续清理精简审批事项规范行政审批中心运行全力打造程序最简、收费最低、效率最高的政务服务。打破市场分割和行政壁垒进一步放开投资领域降低市场准入门槛。依法保护各类市场主体合法权益深入治理涉企乱收费、乱摊派、乱罚款、乱检查严厉打击吃拿卡要、敲诈勒索、欺行霸市、强买强卖等违法活动努力营造良好的经营环境和市场秩序。

(二)、加快新型城镇化进程推进城乡发展一体化。

新型城镇化"一发动全身"。把新型城镇化作为实现转型崛起的重要着力点、统筹城乡发展的重要联结点和改善民生的重要手段坚持扩大规模和提升质量并重推动中心城市、县城、小城镇、新型农村社区协调发展走城乡一体、产城互动、集约节约、生态宜居的新型城镇化道路。

加快构建生态宜居城镇体系。充分发挥南阳的山、水、林、文等优势按照"一圈一副两轴三极"的城镇发展布局推进中心城区与官庄、鸭河、麒麟湖、镇平、社旗、方城、唐河、新野、内乡、南召等组团化发展强化邓州副中心地位促进宁西焦枝铁路、二广沪陕高速两个"十字"形发展轴节点城镇联动发展培育桐柏、西峡、淅川三个发展极。实施中心镇功能提升工程扶持引导基础好的中心镇发展成为小城市。积极稳妥推进新型农村社区建设抓好100个新型农村试点社区的完善提升发展特色产业营造宜居环境建设美丽乡村。

加快中心城区发展。坚持白河为轴、两岸并举、拥河发展、区块突破做大城市做强产业做优形象增强中心城区的承载力、带动力提升中心城市的首位度、宜居度。一是突出规划引领。加强城市发展研究谋划高水准制定完善总规、控规和专项规划以高水平的规划引领城市建设发展。完成城市总体规划报批工作加快推进新型城镇体系规划编制评审尽快完成中心城区控制性单元和景观风貌等专项规划启动编制中心城区郊区总体规划加强城市地下空间的控制和利用。注重内疏外连对内疏通交通、绿廊和绿带逐步整治和疏通内河水系对外进一步连接半小时经济圈促进组团发展。抓紧完善提升中心城区水系规划围绕水系规划与建设统筹安排旧城改造、特色商业街建设集中打造白河轴及其水系网络靓丽的文化和宜居城市名片。强调规划的严肃性严厉打击违规违法建设行为坚决维护规划建设秩序。二是提升建管水平。启动白河南污水处理厂、生活垃圾处理厂、新建续建道路等中心城区城建"十大工程"。加强城市整体形象包装设计和特色景观、重点建筑设计突出城市的雅致之美彰显楚风汉韵特色和山水南阳魅力。完善"大城管"体制健全城市精细化管理长效机制。坚持重心下移构建完善市、区、街道、社区四级城管网络开展"五大延伸"行动实现城市管理全覆盖。启动智慧城市建设数字化城管系统建成投用。实施中心城市"畅通工程"把交通影响评价作为城建项目规划建设的前置条件完善提升交通设施功能建设高效快捷的交通指挥中心系统加快城市主干道交通堵点改造治理启动快速公交专用通道建设。科学规范旧城和城中村改造加快安置房建设。适时启动内河截污整治开发和大水系建设。进一步巩固提升省级卫生城市创建成果争取创建国家卫生城市早日达标。三是壮大中心城区经济。编制实施中心城区经济发展行动计划以先进制造业、现代服务业和高效都市农业为主攻方向着力抓好中心城区三个产业集聚区、四个能源装备制造特色产业园、文化产业园区和商务中心区、特色商业区等增强产业支撑能力。坚持新区开发与老城发展并重激活发展主体实行区块突破。把南阳新区尽快培育成中心城区新的经济增长极。用足、用好国家级高新区平台和政策优势支持高新区打造创新驱动和科技服务示范区培育战略新兴产业高地创建国家创新型特色园区努力实现晋位升级。加强基础设施和公共服务设施建设完善提升东北分区功能。加快官庄、鸭河工区发展确保在基础设施建设、招商项目落地等方面取得突破性进展。进一步明确、理顺市区两级事权鼓励支持宛城、卧龙加快发展。四是加快南阳新区建设。把南阳新区作为推动新型城镇化、发展壮大中心城市的重中之重全力打造现代化复合新城。高水平编制完善新区各项规划。全面实施起步区8大类49个基础设施项目建设年度完成投资80亿元以上。强力推进商务中心区、物流产业区、汽车城等产业项目建设。落实好加快新区建设的决定分领域研究出台支持新区发展的具体政策措施支持新区开展体制机制创新形成推动新区发展的强大合力。

提高城镇化质量。坚持以人为本科学布局强化产业支撑和服务保障推动城市集约节约发展。加快城市新建区域学校、医院、商场等功能性设施建设推动人口快速集聚。建立农村人口有序转移机制降低农业转移人口市民化门槛重点解决户籍、就业、住房、社保、子女入学等问题逐步实现教育、医疗等基本公共服务城镇常住人口全覆盖。把推进城镇化与农业现代化结合起来守住耕地红线保障粮食安全维护农民利益。

加快城乡综合交通运输体系建设。加快建设豫鄂陕接合部区域性交通枢纽。以"一心五环六射二连"为骨架健全完善市域公路网络体系。建设中心城市组团间快速通道。着手启动"二环"路规划建设。加快内邓、三淅、邓州至豫鄂省界高速公路建设做好武西、周南高速公路南阳段前期工作。新建改建干线公路348.2公里完成550公里农村公路改造升级和43座危桥改造任务。合理布局建设客货运场站支持运输场站向物流场站转型。全线启动宁西铁路增建二线工程南阳段建设做好郑万、蒙西至华中等铁路及南阳火车站改扩建工程前期工作。积极推动南阳机场与郑州航空港经济综合实验区的联系互动力争加密至北京、广州航班争取开通北京-南阳-昆明航线。启动南阳机场飞行员训练基地建设。

(三)、加快新型工业化进程构建现代产业体系。

坚持做大总量和优化结构并重促进工业化与信息化、制造业与服务业融合发展实施"大产业、大项目、大企业、大载体"四大工程着力构建以高成长产业为先导以战略支撑产业和现代服务业为主体的现代产业体系。

加快产业集聚区建设。产业集聚区是实现科学发展的主平台是带动城乡统筹发展的有效载体。坚持开放、龙头、项目、创新、品牌、服务六措并举实施"2833"产业集群培育工程研究出台加快产业集群发展专项行动计划建立科学的项目入驻会商评审和考评机制推动各主导产业按照自身特点和发展规律纵向延伸、横向集聚培育竞争优势力争形成3个主营业务收入超百亿元的产业集群。加大产业集聚区基础设施建设力度推动生产性服务功能和公共服务平台建设全面提升综合承载能力。研究出台促进产城互动、以产促城的具体政策措施着力推动产城一体化发展。坚持定期观摩考评推动各产业集聚区比学赶超、加快发展力争全市产业集聚区固定资产投资完成1200亿元以上规模以上工业主营业务收入完成1900亿元以上。

推动工业转型升级。以老工业基地调整改造为契机推动产业产品结构调整打造南阳工业经济升级版。建立"一个产业、一个专班、一个规划、一套政策、一系列支撑载体"的产业发展推进机制力争通过5年努力把装备制造业打造成为千亿级产业培育食品、光电、油碱化工、冶金建材、纺织服装等千亿级后备产业打造中部地区重要的先进制造业基地。运用先进适用技术和现代信息技术改造提升传统产业着力推进战略新兴产业关键技术研发和产业化集中实施100个重大技术改造项目、100个重大核心技术攻关项目、100个重大科技成果转化项目。以优质增量扩充推动工业结构调整抓好108个亿元以上工业结构调整项目全年完成投资155亿元。加快推进河南油田特种蜡生产基地、富士康基地、防爆集团大型防爆电机专业化生产中心、佳吉食品年产100万吨绞股蓝饮料、天冠集团100万吨酶基地、二机防爆公司防爆消防特种车辆、南阳卷烟厂搬迁等对全市具有支撑、带动作用的重大项目。强力推进企业上市加快牧原、玉典钒业、中源化学、中南钻石4家企业上市进程。实施民营企业成长工程和千家中小微企业培育工程培育扶持100家"小强人"企业促进中小企业发展壮大。

搞好经济运行调节。落实稳增长、促回升一系列政策措施帮助企业渡过难关遏制经济下滑势头。开展"企业服务提升年"活动完善企业服务联动、企业问题收集和处理、信息共享、督导考评等工作机制推动企业服务制度化、常态化及时有效解决企业面临的困难和问题。着力开展产销、银企、用工对接做好煤电油气运供需衔接保障企业生产经营需要。

加速发展壮大服务业。健全服务业发展组织协调机构研究出台南阳市现代服务业发展行动计划大力发展新兴服务业改造提升传统服务业提高服务业在生产总值中的比重。一是打造服务业发展载体。商务中心区和特色商业区是实现服务业集聚发展、完善提升城市功能、推动产业转型升级的重要载体。全面完成14个商务中心区和特色商业区空间规划及控制性详规编制。坚持适度超前、统筹协调加快推进商务中心区和特色商业区基础配套设施建设。全力抓好大商集团CBD超大型城市综合体等17个重大项目建设年度完成投资20亿元促进商务中心区和特色商业区尽快出形象、聚人气。高水平策划招商活动在高端商务、文化、旅游、金融等领域引进一批大个头、高质量的项目。二是大力发展商贸物流业。以建设豫鄂陕省际区域性商贸物流中心为目标着力引进、培育商贸物流龙头企业加快推进南阳汇融物流园、美的工业物流园、中商农产品批发市场、中国农批集团农商物流港等项目建设。加快中心城区商业网点规划编制实施和农贸市场建设。三是壮大提升文化、旅游业。集中培育、打造龙头景区实施旅游精品工程引领旅游业提质增效。支持恐龙遗迹园与老界岭、宝天曼与内乡县衙捆绑创建国家5A级景区加快建设渠首生态文化旅游圈和莲花泉国际旅游度假区推进中心城区游客中心、西峡旅游综合体等项目建设。加大宣传推介力度深化南水北调"5+13"区域旅游合作提升南阳旅游知名度力争全年接待游客3500万人次以上实现综合收入180亿元以上。围绕建设文化强市目标推动文化与经济融合发展。加快卧龙岗、张仲景、独山三个文化产业集聚区和南阳现代中药产业园规划建设步伐抓好南阳卧龙国际玉文化博览中心、国际玉城二期、社旗县文化展演中心等项目建设着力推动镇平玉文化改革发展试验区申报创建国家级试验区。借助文化、旅游业壮大提升带动酒店宾馆餐饮服务业稳定发展。四是加速壮大金融业。高水准规划建设金融产业集聚区。争取设立南阳农村金融综合改革试验区。积极引进广发银行等金融机构入驻我市推动南阳村镇银行向重点乡镇延伸加快县市区农信社组建农村商业银行步伐。支持南阳银行引进战略投资者尽快做大做强创建地方金融品牌。积极发展证券、保险、信托、担保等金融业务。拓展融资渠道新增融资规模200亿元。建立完善金融风险防控和社会信用体系营造良好的金融生态环境。

(四)、加快新型农业现代化进程强化农业基础地位。

始终把"三农"工作放在重中之重的位置以"保粮食、调结构、促增收、惠民生"为中心任务大力发展现代农业。一是抓好粮食生产保障国家粮食安全。围绕到2020年建成800万亩高标准粮田目标加快推进高标准粮田"百千万"工程建设今年确保完成130万亩建设任务市级重点抓好30万亩示范区规划建设各县市区重点抓好一个万亩粮食示范方。开展高产创建活动推广吨粮田技术集成模式确保粮食总产达到120亿斤以上。二是大力发展特色农业培育壮大农业产业化集群。充分发挥我市作为部分农产品原产地和主产区的优势尽快上规模、建标准、创品牌。大力发展草腐菌、猕猴桃、肉牛、蔬菜、苗木花卉、中药材、茶叶等高效特色富民产业。因地制宜发展休闲观光农业、园区农业等新兴农业。在每个县市区打造一个万亩高效农业示范区、一个标准化畜牧养殖示范区。着力培育20个农业产业化集群重点抓好新野肉牛、牧原生猪、西峡食用菌、宛西制药、社旗食品加工、河南三源粮油等农业产业化集群建设力争销售收入增长15%以上。把打通一二三产业、形成优势品牌和拉长产业链条作为提高农业比较效益的主攻方向积极培育壮大龙头企业加快发展新型农村合作组织和农业经营性服务组织扶持发展联合经营、专业大户和家庭农场完善"公司+基地+合作组织+农户"利益联结机制促进土地适度规模经营着力构建集约化、专业化、组织化、社会化的新型农业经营体系。同时注意保护法律赋予农民的财产权利调动农民积极性维护农村稳定。三是完善农业支撑体系。持续实施农业"三百"工程和农业科技"三十"工程。健全农业(农机)技术推广、动植物疫病防控、农产品质量安全监管等公共服务机构建立完善重大自然灾害监测预警系统、农产品检验检测和追溯体系全面启动县级农产品市场准入制度。加快推进农业机械化进一步夯实现代农业发展物质基础。建设一批区域性农产品批发交易市场继续实施新农村现代流通网络工程建设支持供销社、大型物流集团开展农产品流通业务。四是加强农业基础设施建设。加快鸭河口、引丹、宋岗三个大型灌区续建配套和石步河水库建设进度完成20个中小河流治理、138座病险水库除险加固任务加快推进唐白河防洪治理二期工程前期工作。抓好农综开发、土地整理和11个小型农田水利重点县项目建设。

(五)、加快生态文明建设打造美丽南阳。

加快生态文明建设既是服务保障南水北调中线工程建设的政治责任也是增创南阳发展新优势的现实选择。牢固树立尊重自然、顺应自然、保护自然的生态文明理念重视和珍惜南阳良好的生态环境条件推进国家生态文明试点市规划建设实行生态建设与环境保护一票否决制度努力实现绿色发展、循环发展、低碳发展加快构建"两型"社会打造美丽南阳。一是优化国土空间开发格局。尽快编制实施我市主体功能区规划根据区域资源禀赋、环境承载能力明确划分生态保护功能区、生态农业功能区和产城互动功能区建立差异化的考核体系推动各地严格按照主体功能定位发展牢牢守住1488万亩耕地"红线"、1630万亩林地"绿线"和400万亩湿地水域"蓝线"。二是加强生态建设和环境保护。突出山、水、气、田等要素着力构建以"两山两水"为重点的生态战略格局强化环境综合整治打造天蓝地绿、山清水秀、生态宜居的美好家园。加强山体绿化和生态廊道建设大力开展荒山绿化和矿山整治复绿加快推进白河生态景观林带和以高速公路为主的通道绿化等林业生态重点工程全年完成营造林121.3万亩新建省级以上森林公园、湿地公园4个。优化水生态环境落实最严格的水资源管理制度编制实施全市河流水系生态保护和水污染防治规划加强水源涵养和水土保持实施河流廊道生态系统保护与修复加大水污染防治力度加快污水处理厂等城乡环保基础设施建设开展鸭河口水库等重点水源区和白河等骨干河流水质监测保护加快城市内河治理构建水清岸绿的水生态环境。加大大气污染防治力度开展扬尘、机动车尾气和工业废气治理积极推进电力、水泥、钢铁等行业脱硫、脱硝推广应用天然气、沼气等清洁能源启动中心城区PM2.5检测努力提高空气质量。建设清洁田园狠抓农村面源污染治理推广高效低毒低残留农药、生物防治技术和测土配方施肥控制农药和化肥过量使用加强农用废弃物管理夯实生态农业和食品安全基础。提高资源集约节约利用水平推行国内领先的能耗、物耗、水耗标准强化各类园区建设用地投资强度及标准控制。三是发展生态经济。积极探索生态经济化、经济生态化的发展路子努力实现经济、生态、社会效益统一。发展生态工业实施一批循环经济重大工程和项目重点推进纤维乙醇规模化生产、二氧化碳综合利用、碱硝化工循环经济示范区等项目。培育生态旅游业推动我市中医药等休闲养生产业与旅游业融合发展加快麒麟湖等休闲养生基地建设打造休闲养生之都。推广淅川、西峡发展生态农业的成功经验走出一条生态植被作物化、经济作物产业化的生态经济化之路。四是做好南水北调中线工程建设服务保障。今年是南水北调中线工程三年攻坚的决战之年服务保障和配套工程建设任务十分繁重。深化"双创双评"活动争创全线最优施工环境。全力推进配套工程、库底清理和农村外项目建设。加快南水北调中线工程渠首区地震应急指挥、应急避难场所等项目建设。加快编制实施《渠首水源地高效生态经济示范区林业规划》和《南水北调中线生态走廊规划》高标准打造南水北调中线生态走廊。深入实施《丹江口库区及上游水污染防治和水土保持"十二五"规划》全面开工建设丹江口库区水质保护项目加快渠首环境保护中心建设推进"环农工程"编制实施一河一策水污染防治方案持续做好水质保护工作。

(六)、加快实施创新驱动战略激发发展活力。

加快国家创新型试点城市建设增强经济发展的内生动力和活力。一是推动科技创新。突出企业主体。充分发挥政府在科技发展中的引导支持作用围绕"四个领域一抓手"坚持自主创新与借力创新并举确立企业在资金投入、科研组织、技术研发、成果转化全环节的核心地位。完善结合机制。构建以企业为主体、市场为导向、产学研结合的协同创新体系。积极推动光电、新能源、装备制造等产业建立技术创新联盟。支持企业申报国际科技合作对欧对俄专项、防爆集团建设国际科技合作基地。加快实施南阳市--中关村科技园区战略合作等国家创新型城市建设重点项目。扩充创新载体和研发平台。充分发挥产业集聚区的综合载体作用实施产业集聚区科技创新工程。支持南阳新能源产业集聚区建设省级创新型产业集聚区。推动重点企业建立研发机构争创省级以上工程技术中心和重点实验室。以推动卧龙国家级农业科技园区达标验收为抓手加快农业领域科技创新步伐。高标准建设一批国家级、省级检测中心。筹建南阳市产品质量安全综合检测基地积极申报国家级特种车辆质检中心建成国家级粮油检测中心加快建设省级天然碱、碳酸钙检测中心。完善创新服务支撑体系。加快南阳市自主创新公共服务平台建设。落实激励企业自主创新的优惠政策。设立科技创新引导资金加快形成多渠道的科技资金投入体系力争全社会科技研发投入占生产总值的比重达到1.5%。积极创建国家知识产权示范市。持续推进质量提升、标准化和品牌带动健全完善市长质量奖评选制度。争取再有1家企业获得省长质量奖创建国家级品牌20个省级品牌100个以上争取国家、省重大科技项目5项争创省级研发中心7个。二是深化改革创新。加速推进投融资、行政审批、集体林权、厂办大集体、财政预算绩效管理和国库集中收付等经济领域改革创新加大事业单位分类改革、人事工资制度等社会领域改革力度。加快推进农村集体土地所有权、土地承包经营权、宅基地使用权确权登记颁证工作持续抓好农村综合改革。三是大力推动全民创业。培育壮大民营经济。广泛开展"全民创业行动"完善落实激励创业的各项优惠政策加强创业培训树立创业典型鼓励引导大中专毕业生、退伍军人、下岗职工、返乡农民工等自主创业力争全年发放小额担保贷款6亿元扶持创业1.2万人。认真贯彻落实国家促进民营经济发展的36条意见等政策措施拓宽社会资本进入领域。关心、爱护、支持企业家建立完善有利于企业家发展的良性机制使企业家成长有平台、队伍有梯次、创业有政策、政治有地位、安全有保障。四是实施人才强市战略。围绕经济发展重点领域区域致力培养开发引进高技能人才建设一支高素质人才队伍。加快博士后科研工作站等引才引智主体建设畅通人才引进"绿色通道"深入实施专业技术人才知识更新工程和引进国外智力工程。创新大中专院校、科研院所的人才培养机制。实施全民技能振兴工程和"急需紧缺高技能人才培养计划"进一步健全高校、职业学校与产业园区的"校企合作"模式全年培训各类人才17.5万人次。创新人才评价机制加大奖励力度做好服务保障逐步实现从就业引才到创业聚才的转变。

(七)、切实保障和改善民生提升和谐社会建设水平。

顺应人民群众对美好生活的期待继续加大投入实施"十项重点民生工程"着力解决人民群众最关心、最直接、最现实的问题。一是着力促进就业提升社会保障水平。实施更加积极的就业政策加强基层就业服务平台和创业带动就业孵化基地建设多渠道开发就业岗位做好用工对接推动实现更高质量的就业。新增城镇就业9万人农村劳动力转移就业8万人高校毕业生就业率稳定在85%以上城镇登记失业率控制在4.5%以内零就业家庭动态归零。坚持"广覆盖、保基本、多层次、可持续"的方针建成覆盖城乡居民的社会保障体系。实施社会保险扩面征缴行动实现各类社保参保人员累计达到931万人次以上。理顺社会保障管理经办体制实现社会保障"一卡通"。及时调整城镇企业退休人员基本养老金和城乡居民基础养老金标准加快推进养老服务体系建设。适当提高居民医保财政补助和个人缴费标准。提升城乡低保、农村五保供养标准和优抚对象补助水平。完善被征地农民社会保障政策。完成13万农村人口稳定脱贫任务。二是大力发展社会事业。办好人民满意的教育。坚持教育优先发展战略推进各级各类教育协调均衡发展全面提升教育质量努力打造教育强市。整合各类教育资源优化教育布局加速城镇中小学校和学前教育扩容提质中心城区新建、改扩建一批中小学校和幼儿园强力推进一高中新校区建设尽快缓解"大班额"、"入学难"等问题。扶持引导农村教育健康发展逐步改善办学条件。加大职教园区建设力度加快南阳农业职业技术学院建设全力推进张仲景医学院筹建工作启动南阳职业学院建设支持南阳幼师升专。强化校地合作提升高校建设水平。支持南阳理工学院顺利通过教育部本科教学工作合格评估。提高人民健康水平。深化医药卫生体制改革优化卫生资源配置切实缓解群众看病难、看病贵问题。加快卫生信息化建设积极稳妥推行"先诊疗后结算"模式。新农合人均筹资水平提高到340元。抓好全科医生培训基地等4大类25个项目建设。大力发展中医药事业统筹建设市县乡中医院三级体系。扶持村级卫生室建设。深化全民健身示范市创建成果办好市第四届运动会加快方城、南召、西峡等地体育场馆建设积极做好后农运时代体育场馆的开发利用。坚持计划生育基本国策提高出生人口素质促进人口长期均衡发展。提升文化服务能力。把文化改革发展纳入经济社会发展总体规划。抓紧开展"三馆一院一店"和中国张衡地震科普文化馆项目前期工作。把历史文化遗产保护纳入城市规划建设总体布局完成武侯祠、香严寺、内乡县衙等文物保护单位的保护维修工程。继续抓好文化惠民工程博物馆、纪念馆免费开放达到15个。精心组织开展"欢乐南阳"群众文化活动。制定促进文艺精品创作生产扶持办法繁荣文艺创作。三是致力改善城乡居民生活条件。把食品、饮水、住房等人民群众关注的热点问题摆到更加突出的位置来抓努力让人民群众吃上安全食品喝上洁净水呼吸上清新空气过上更加幸福的生活。改革和健全食品药品监管体制机制严格从生产源头到消费的全程监督严厉打击食品安全违法行为提升食品药品安全保障水平。年内实现城市集中式饮用水源地水质监测全部达标新解决25万人以上农村居民和学校师生饮水安全问题。认真贯彻落实国家房地产调控政策加大普通商品住房供应抑制房价过快上涨。扩大住房公积金制度覆盖面。开工建设各类保障性住房2万套。完成农村危房改造1.5万户。扎实推进移民后续稳定发展工作落实移民扶持政策开展移民增收致富竞赛和文明新村(社区)、移民户创建活动完成总投资2亿元的移民扶持项目和2万人次移民就业技能培训。四是加强和创新社会管理。增强城乡社区服务功能完善村(居)民自治制度健全社会管理服务网络。全面推行劳动合同制度和集体合同制度推进仲裁机构实体化加强劳动保障守法诚信建设着力构建和谐劳动关系。建立减轻农民负担的长效机制。畅通群众诉求表达、利益协调、权益保障渠道切实做好信访工作。加强应急管理不断提高突发事件应对处置能力。加强安全生产管理深化平安南阳、法治南阳建设强化社会治安综合治理持续开展严打整治行动确保社会大局和谐稳定。

继续加强国防动员、国防教育和国防后备力量建设增强全民国防观念支持驻宛解放军、武警消防部队、预备役部队、民兵和人民防空建设扎实开展军民共建活动巩固全国双拥模范城创建成果。大力加强精神文明建设用好"编外雷锋团"等载体发挥好王金山等英模人物的群体表率作用以道德风尚引领社会潮流。认真做好全国第三次经济普查。支持工会、共青团、妇联、工商联等人民团体发挥桥梁纽带作用。继续做好审计、外事侨务、对台、民族宗教、统计、史志、档案、气象、地震、驻外办事机构等工作。

折叠全面加强政府自身建设

在新的起点上实现南阳绿色崛起是全市人民的殷切期盼更是各级政府的历史使命和重大责任必须加倍努力、不懈奋斗。我们要坚持既定战略不动摇一张蓝图绘到底深入推进"两转两提"努力建设为民务实高效清廉、人民满意的政府。

(一)、提高施政水平。以政府系统执政能力建设为核心不断提高政府谋划、决策、执行能力。树立全局意识和战略思维深化细化研究把握运用规律准确研判形势注重战略谋划强化策略设计健全决策机制和程序推行科学决策、民主决策。稳妥推进政府机构改革和职能转变理顺健全政府职责体系优化工作流程推行绩效考评严格行政问责提高政府执行力和行政效能。拉高标杆提高标准落实责任加快工作节奏又好又快推进各项工作。健全重点工作和项目分包责任制、专班推进、联席办公、现场观摩、目标督查等机制做到有部署、有督促、有奖惩、有必要的事后评估确保各项决策和目标任务落到实处。

(二)、严格依法行政。严格依照法定权限和程序行使权力、履行职责、规范行为自觉用法治思维和方式调节经济运行、管理社会事务。严格规范公正文明执法强化行政执法监督检查规范行政处罚裁量权坚决查纠执法不力、执法违法、执法扰民、执法寻租等行为。深化政务公开公开部门"权力清单"。自觉接受人大及其常委会的法律监督、工作监督和政协的民主监督坚持向人大报告工作向政协通报情况认真办好人大代表建议和政协委员提案。广泛听取民主党派、无党派人士、人民团体意见虚心接受社会监督和舆论监督。

(三)、切实转变作风。严格执行中央和省、市关于改进工作作风、密切联系群众的八项规定、若干意见和十条规定认真开展党的群众路线教育实践活动。完善干部直接联系群众制度问政于民、问需于民、问计于民。坚持求真务实勇于担当恪尽职守负重拼搏。改进调研方式简化接待应酬精简会议、文件减少检查评比力戒形式主义、官僚主义。开展整治懒庸散专项活动强化政风行风评议探索实施电视问政提高工作效率和服务水平营造良好的政务环境。积极开展"人民满意的公务员和公务员集体"评选活动确立"以业绩论英雄、无功就是过"的价值导向持续弘扬敢于担当、比学赶超的干事创业、争先创优之风。

(四)、坚持廉洁从政。认真落实党风廉政建设责任制全面推行惩治和预防腐败体系建设健全权力运行制约和监督体系。深入开展各类专项整治活动加强对重点领域和关键环节的监督。加强对领导干部的经济责任审计。充分发挥公共资源交易中心功能进一步规范招投标、产权交易、土地使用权出让、政府采购等工作。坚持勤俭办一切事业反对奢侈浪费严控行政经费等一般性支出严控楼堂馆所建设。加大违法案件查处力度以反腐倡廉的实效树立形象、取信于民。

折叠编辑本段答谢

各位代表!实干成就伟业奋斗铸就辉煌。让我们在省委、省政府和市委的坚强领导下凝心聚力奋发有为用我们的勤奋和智慧共同创造南阳更加美好的未来!
